# Study on the Residence Time and Texture Prediction of Pea Protein Extrusion Based on Image Analysis

**DOI:** 10.3390/foods12244408

**Published:** 2023-12-07

**Authors:** Qi Wu, Xun Zhang, Fei Gao, Min Wu

**Affiliations:** 1 Institute of Collaborative Innovation, University of Macau, University of Macau Avenida da Universidade Taipa, Macau 519000, China; mc25587@connect.um.edu.mo; 2 College of Engineering, China Agricultural University, P.O. Box 50, No. 17 Qinghua East Road, Haidian District, Beijing 100083, China; zhangxun@cau.edu.cn; 3 School of Food and Health, Beijing Technology and Business University, No. 11 Fucheng Road, Haidian District, Beijing 100048, China; feigao@btbu.edu.cn

**Keywords:** extrusion texturization, neural network, prediction model, Residence Time Distribution, texture quality

## Abstract

This paper initially involves three main processing parameters: screw speed, feeding speed, and initial material moisture content, exploring the RTD of materials inside the extruder barrel under varying parameters and clarifying the impact of parameter variations on RTD. Finally, machine vision technology was utilized to link extruded product images to texture features, and a texture prediction model based on image features was established using a Back Propagation (BP) neural network. Particle Swarm Optimization (PSO) and Genetic Algorithm (GA) were applied to optimize the BP neural network. The results indicate that the feeding speed has a stronger impact than the screw speed on the extrusion process, and an increase in the initial material moisture content tends to shorten the RTD. Specifically, an increase in screw speed results in a denser product structure, while higher feeding speeds lead to reduced pore size in the microstructure. As the initial material moisture content increased from 55% to 70%, the average residence time MRT decreased from 265.21 s to 166.62 s. Additionally, elevated moisture content causes a more porous microstructure. After optimizing the texture prediction model of extruded products through the application of Particle Swarm Optimization and Genetic Algorithm models, it was discovered that the Genetic Algorithm was more effective in reducing errors (*p* < 0.05) than the Particle Swarm Optimization algorithm. It was found that the Particle Swarm Optimization model exhibited better prediction performance. The results of the prediction indicated a significant association between the image features of the product and hardness, resilience, and chewiness, as corroborated by correlation coefficients of 0.93913, 0.94040, and 0.94724, respectively.

## 1. Introduction

With the rapid growth of population in modern society, the supply of meat relying solely on animal husbandry will be unable to meet the needs of human daily life. At the same time, environmental pollution, antibiotic and hormone residues, viral infections, and other problems brought about by the animal husbandry industry are also threatening food safety, so it is imperative to find effective meat substitutes; plant proteins have become an effective way to find ways to reduce the consumption of meat products without lowering the amount of protein intake. According to statistics, plant proteins account for more than 80% of the world’s total protein production, and legumes, grains, and oil crops are rich in high-quality proteins. Compared with animal proteins, plant proteins are more easily absorbed by the human body, and their production requires far fewer resources and causes less pollution and environmental damage than animal proteins. Due to its wide availability and rich nutrients, plant protein is increasingly considered as one of the viable alternatives to animal protein in meeting the growing demand for protein in humans. Pea protein, as a full-value plant protein that is resourceful, nutritious, and cholesterol-free, and able to replace animal protein to meet the growing demand for protein, is the best choice for human beings. Extruded tissue processing is an important means of making plant protein with the taste and texture of animal meat. Processing plant protein using extrusion techniques can be a crucial method to impart meat-like texture and mouthfeel to plant-based protein products. The texture features of extruded products are an important indicator of the extrusion process. Traditional methods for creating texture features are time-consuming, labor-intensive, and often result in sample structure damage, with relatively poor accuracy. However, the establishment of a product texture prediction model based on image features using neural networks provides a feasible approach for the rapid, non-destructive, and precise detection of sample texture characteristics.

The extrusion process of a twin-screw extruder is a complex process that combines physical, chemical, and biological reactions. The “black box” processing characteristic of the extruder barrel makes the dynamic processing of materials ambiguous, making it challenging to understand the flow status of materials within the barrel. Residence Time Distribution (RTD) is one of the critical parameters during the extrusion process, representing the duration of time from the moment materials enter the barrel until the extruded products exit the cooling die head [1]. Due to the high temperature and pressure in the barrel, the molten materials exhibit certain differences in mixing and flow status, resulting in variations in residence times even for materials that simultaneously enter the extruder. Therefore, exploring the material RTD within the barrel can provide a better understanding of their mixing and flow status, which has significance for the quality control of the extruded products.

In the process of plant protein extrusion texturization, the quality characteristics of the extruded products are the most crucial evaluation indicators, primarily realized through sensory evaluation and physical property testing [2,3]. Sensory evaluation is relatively subjective, with product color, texture, flavor, and taste being the main indicators [4]. Physical property testing mainly relies on texture analyzers and universal material testing machines by calculating product hardness, chewiness, and texturization [5]. To obtain the texture characteristics of the tested samples, Texture Profile Analysis (TPA) is conducted using a texture analyzer, which uses two compression tests to simulate human mastication. TPA causes irreparable damage to the sample structure, with the testing process being time-consuming and labor-intensive. Color is another important sensory characteristic of the extruded samples [6] which is closely related to their textural features [7,8].

A Back Propagation (BP) neural network is an information processing system capable of handling complex issues, with prediction being one of its most crucial functions. It features a strong self-learning ability, robust adaptability, and extendability, and has been widely applied across various industries [9,10,11,12]. BP neural networks break the conventions of linear prediction, exhibiting robustness especially in the prediction of multi-input nonlinear systems and models [13]. By capturing images of the extruded samples, extracting relevant image features, and combining with BP neural networks for model construction, it is possible to achieve non-destructive, rapid, and efficient inspection of textural features. BP neural networks can translate complex real engineering problems into simple mathematical model issues [14], with trained networks providing prediction results for given inputs under well-trained predictive models, establishing the correlation between inputs and outputs [15]. However, BP neural networks also tend to fall into local optima, leading to poorer predictive model effects. Currently, the optimization of neural networks is primarily achieved to attain the optimal prediction model. Particle Swarm Optimization (PSO) and Genetic Algorithm (GA) are common model optimization methods [16] which are both capable of ameliorating situations in which BP neural networks can easily fall into local optima, thus achieving optimal prediction effects [17,18].

This paper aims to determine the RTD under different extrusion processing parameters through pea protein extrusion experiments using the pulse method, a more convenient and efficient imaging method that replaces the traditional colorimetry method. By altering the screw speed, feeding speed, and initial material moisture content, the impact of changes to extrusion processing parameters on the mixing and flow status of pea protein within the extruder barrel was explored, and the influence of screw speed, feeding speed, and initial material moisture content on material RTD was analyzed. Based on this, a BP neural network combined with PSO and GA optimization algorithms was employed to explore the optimization effects in the texture characteristic prediction model based on the extruded product images.

## 2. Materials and Methods

### 2.1. Materials

Pea protein was supplied by Yantai Shuangta Food Co., Ltd. (Yantai, China) with 85% protein concentration and 7% moisture content. Erythrosine B Sodium Salt with 85% purity was acquired from Shanghai Macklin Biochemical Co., Ltd. (Shanghai, China).

### 2.2. Pea Protein Extrusion Texturization Experiment

This experiment was conducted with the co-rotating twin-screw extruder (TwinLab-F 20/40, Brabender, Oberhausen, Germany) experimental platform. This platform primarily consists of a power input unit, an extrusion shearing unit, a temperature and pressure monitoring unit, a cooling circulating water unit, and a water and material feeding unit. Its main technical specifications include five independent heating zones, outer screw diameter of 20 mm, inner screw diameter of 12.5 mm, length-to-diameter ratio of 40:1, maximum power of 10 kW, screw speed range of 0–1200 rpm, maximum torque of 2 × 40 Nm, working temperature range of 0–400 °C, and maximum production efficiency of 20 kg/h.

A regression relationship was established between the set values and actual values of the water and material feeding units by setting the water feeding speeds at 20 rpm, 40 rpm, 60 rpm, 80 rpm, and 100 rpm, and the material feeding speeds at 10 rpm, 20 rpm, 30 rpm, 40 rpm, and 50 rpm, measuring the actual water and material feeding rates, repeating this 3 times, and establishing a regression equation.

**Figure 1:**
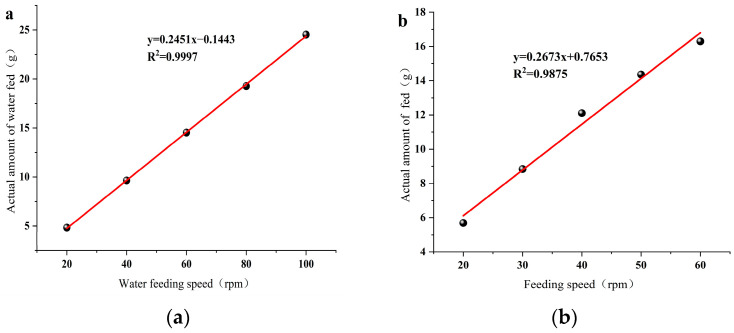
Regression relationship between wat-er feeding, material feeding speed, and actual speed. (**a**) represents the relationship between water feeding speed and actual speed; (**b**) represents the relationship between material feeding speed and actual speed.

Figure 1 demonstrates that there is a high correlation between the set values and actual values of the water and material feeding units, with high accuracy. The correlation coefficients R^2^ reached 0.9997 and 0.9875, respectively. Based on this, the ratio relationship between the speeds of the material feeding unit and water feeding unit were obtained with the following formula.

(1) $$V_{\mathrm{wf}}(g/\min)=\frac{C_{ex}\;(\%)-\;C_{in}\left(\%\right)}{\;1-C_{ex}\left(\%\right)}\times V_{mf}\left(g/\min\right)$$

where “*V*_wf_” represents “Water Feeding Speed”, “*V_mf_*” represents “Material Feeding Speed”, “*C_ex_*“ represents “ Expected Material Moisture Content”, and “*C_in_*“ represents “ Initial Material Moisture Content”.

After starting the extruder and preheating it, the water feeding was initiated once the temperature reached the preset value and was stable. Upon water flow from the outlet, the feeder was activated along with a coordinated adjustment of the water feeding speed. When material extrusion was observed at the outlet, the screw speed, material feeding speed, and initial material moisture content were gradually adjusted to ensure stability throughout the foundational process before proceeding with sampling or other operations.

### 2.3. Pulse Method Residence Time Determination

Improvements were made on the traditional pulse method, with food-grade dye Erythrosine B Sodium Salt, featuring bright color, high stability, and strong coloring, being selected as the indicator. The specific operation procedure for the RTD measurement experiment was as follows: Once the extruder ran smoothly and stably and output samples, the feeder was lifted, 0.05 g of Erythrosine B Sodium Salt was instantaneously added, and timing began (t = 0), while extruded samples were simultaneously collected from the extruder every 10 s, recording the time, until no red color was observed in the extruded samples, at which point collection ceased.

A total of 2.2 kg of pea protein raw material was equally divided into 11 equal parts, each weighing 200 g. Erythrosine B Sodium Salt tracer was added to create standard samples within the concentration range of 0–0.4 mg/g, following a concentration gradient of 0.04 mg/g, and thoroughly mixed using a small powder mixer (33 rotations/minute, 15 min) for preparation. The extrusion conditions were as follows: initial material moisture content of 65%, material feeding speed of 35 rpm, water feeding speed of 125.8 rpm, and screw speed of 100 rpm. The set temperatures for the five independent heating zones were 50 °C, 70 °C, 90 °C, 110 °C, and 110 °C.

With the extrusion parameters set, sampling commenced according to the aforementioned experimental method. Between every two groups of different concentration materials, 200 g of pea protein raw material was added as a blank control to eliminate the interactive impact of the indicator on the previous group of materials.

### 2.4. Sample Color Feature Extraction by Conventional Methods

The extruded samples of pea protein extrusion texturization obtained by mixing different concentrations of indicator were collected, naturally cooled to room temperature, pre-frozen in a −4 °C refrigerator for 12 h, and then vacuum freeze-dried in a vacuum freezing drying (LGJ-18C, Si-Huan, Beijing) oven for 36 h. After freeze-drying, the samples were crushed using a high-speed pulverizer and sieved through an 80-mesh sieve to obtain the extruded sample powder. The color difference of the sample powder was measured using a colorimeter (Labscan XE, HunterLab, Reston, VA, USA), with each sample group measured three times and the average value taken.

### 2.5. Experiments on the Effect of Residence Time of Pea Protein

The temperatures of the five independent heating zones inside the extruder barrel were set to 50 °C, 70 °C, 90 °C, 110 °C, and 110 °C. The experiment was conducted with screw speed, material feeding speed, and initial material moisture content as the single-factor variables. For the purpose of the experiment, three different levels of screw speed were used: 70 rpm, 100 rpm, and 130 rpm. Similarly, three levels of material feeding speed (25 rpm, 30 rpm, and 35 rpm) and four levels of initial material moisture content (55%, 60%, 65%, and 70%) were employed.

### 2.6. Residence Time Distribution (RTD)

The Residence Time Distribution function, E(t), represents the curve delineating the variation in tracer concentration within the extruded sample over the extrusion time. This is essentially the ratio of the tracer concentration at various instances to the total concentration of the tracer within the extruded samples.

(2) $$E(t)=\frac{C(t)}{\int_{0}^{\infty }C(t)\mathrm{dt}}=\frac{C_{i}}{\sum_{0}^{n}C_{i}\Delta t_{i}}\;$$

where “C(t)” represents the concentration of the tracer in the extruded sample at the time “t”, “C” “i” represents the concentration of the tracer in the segment “i”, and “t” “i” represents the extrusion time in the segment “i”.

The Cumulative Residence Time Distribution function, F(t), is derived from the E(t) curve calculations. The F(t) curve characterizes the flow state of the material within the extruder barrel, illustrating the temporal variation in the accumulated amount of indicator concentration at the extruder outlet. It represents the area between the E(t) curve and the time axis before a certain t moment. For ease of analysis and comparison, the F(t) curve is typically represented using normalization processing.

(3) $$F(t)=\int_{0}^{t}E(t)\mathrm{dt}=\frac{\int_{0}^{t}C(t)\mathrm{dt}}{\int_{0}^{\infty }C(t)\mathrm{dt}}=\frac{\sum_{0}^{t}C_{i}\Delta t_{i}}{\sum_{0}^{n}C_{i}\Delta t_{i}}$$

where “C” “i” represents the concentration of the tracer in the extruded sample at the time “t” “i”, “t” “i” represents the extrusion time in the segment “i”, and “n” is the total number of sampling times.

The average residence time MRT and variance σ^2^ were obtained by plotting the obtained E(t) and F(t) values against time, by which the distribution pattern of the material’s residence time in the barrel can intuitively and qualitatively obtained, and the material’s residence time in the barrel can be quantitatively described with greater accuracy.

(4) $$\mathrm{MRT}=\frac{\int_{0}^{\infty }\mathrm{tE}(t)\mathrm{dt}}{\int_{0}^{\infty }E(t)\mathrm{dt}}=\int_{0}^{\infty }\mathrm{tE}(t)\mathrm{dt}=\frac{\int_{0}^{\infty }C(t)\mathrm{tdt}}{\int_{0}^{\infty }C(t)\mathrm{dt}}=\frac{\sum_{0}^{n}C_{i}t_{i}\Delta t_{i}}{\sum_{0}^{n}C_{i}\Delta t_{i}}$$

(5) $$\sigma^{2}=\int_{0}^{\infty }t^{2}{E(t)\mathrm{dt}-\mathrm{MRT}}^{2}=\frac{\int_{0}^{\infty }{C(t)t}^{2}\mathrm{dt}}{\int_{0}^{\infty }C(t)\mathrm{tdt}}{-\mathrm{MRT}}^{2}=\frac{\sum_{0}^{n}C_{i}t_{i}{}^{2}\Delta t_{i}}{\sum_{0}^{n}C_{i}\Delta t_{i}}{-\mathrm{MRT}}^{2}$$

where “C” “i” represents the concentration of the tracer in the extruded sample at the time “t” “i”, “t” “i” represents the extrusion time in the segment “i”, and “n” is the total number of sampling times.

Due to the multiple physical field factors affecting the extrusion process, the obtained “E(t)” curves and “F(t)” curves were difficult to quantitatively compare under different extrusion conditions and actual situations. Therefore, a dimensionless time ”θ” was introduced to replace the original actual time t, and was used to compare the distribution patterns and flow state conditions under different circumstances.

(6) $$\theta =\frac{t}{\mathrm{MRT}}\;\;\;$$

(7) E (θ)=MRT×E(t)

(8) F (θ)=F(t) 

(9) $$\sigma_{\theta }^{2}=\frac{\sigma^{2}}{{\mathrm{MRT}}^{2}}\;$$

There are two classic flow types in fluid flow: plug flow and mixed flow. In the fluid flow process, the fluid can only flow forward without axial mixing; this laminar flow mode is called plug flow. Mixed flow indicates that the fluid has undergone sufficient mixing during the flow process, as not only has forward flow occurred, but the fluid has also undergone a certain amount of axial mixing. Mixed flow is a more ideal flow state in fluid research.

The mixing state of the material in the extruder barrel is represented by the Peclet number (Pe), which is used to describe the degree of material dispersion. The Peclet number (Pe) is defined as the axial dispersion distance. The Pe value can be derived by extrapolation from Equations (4) and (5) [19]. The larger the Pe value, the greater the degree of material dispersion, indicating that the material flow is plug flow. Conversely, the smaller the value, the more a flow tends to be a mixed flow, with a better mixing effect of the material, which can effectively improve the quality of extruded products.

(10) $$\sigma_{\theta }^{2}=\frac{2}{\mathrm{Pe}}-\frac{2}{{\mathrm{Pe}}^{2}}{\;(1-e}^{-\mathrm{Pe}}\;)\;$$

### 2.7. Microstructure Measurement of Pea Protein Extrusion Products

The pea protein extrusion texturization experiment was conducted separately with screw speed, material feeding speed, and initial material moisture content as the single-factor variables. When screw speed was used as the single-factor variable, the experiment was conducted at screw speeds of 70 rpm, 100 rpm, and 130 rpm, based on a fixed material feeding speed of 30 rpm. When material feeding speed was used as the single-factor variable, the experiment was conducted at material feeding speeds of 25 rpm, 30 rpm, and 35 rpm, based on a fixed screw speed of 100 rpm. When initial material moisture content was used as the single-factor variable, the experiment was conducted at initial material moisture contents of 55%, 60%, 65%, and 70%, based on a fixed screw speed of 100 rpm and a material feeding speed of 30 rpm. The materials obtained from extrusion were observed for the microstructure of the extruded freeze-dried pea protein texturization products using a Scanning Electron Microscope (S-3400N, Hitachi, Ltd. Tokyo, Japan). The dried samples were brittle-fractured, and fragments from the fractured section were selected. The samples were adhered to the SEM sample tray with conductive glue, gold-sprayed for 1 min in a vacuum environment, and electron micrographs of the samples magnified 500 times were obtained under an accelerating voltage of 15.00 kV.

### 2.8. Experiments on Texture Feature Prediction Based on Neural Network

**Figure 2:**
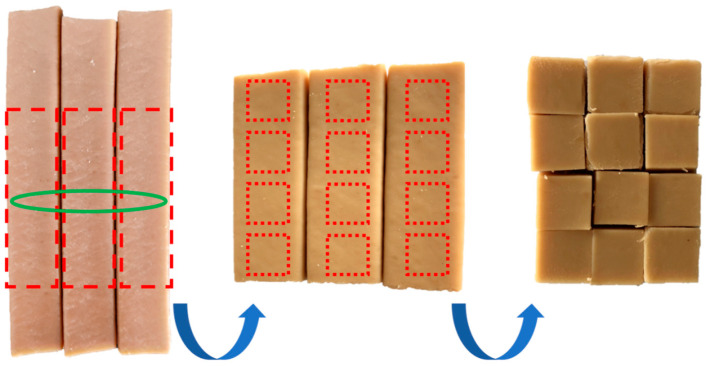
Sample collection and processing flow chart.

For this experiment, the screw speed was set at 100 rpm, the material feeding speed at 35 rpm, the initial material moisture content was adjusted to 60%, and the cooling die head temperature was set at 40 °C ± 1 °C. Extrusion temperature was taken as the single-factor variable, adjusting the temperatures of the five independent heating zones in the extrusion barrel. The temperature for the solid conveying section was 50 °C, the mixing section was 70 °C, the cooking section was 70 °C, and the temperatures for the melt conveying section were set at a gradient of 70–80–90–100–110–120–130–140–150–160 °C across 10 groups. Once stable samples were extruded from the extruder, three sections of extruded samples of equal length were continuously taken. From the same position in the three sections, three sections of equal-length samples were cut out, and each section was further divided into four pieces (1 cm × 1 cm) of final samples using a cutter, as shown in Figure 2. The temperature for the melt-conveying section was adjusted from 70 °C to 160 °C in sequence for sampling, with the sampling conditions kept consistent, from which 10 groups of final samples were obtained.

### 2.9. Collection of Sample Images and Texture Measurement

The collected extruded test samples were sequentially subjected to texture TPA (Texture Profile Analysis) mode measurement according to the labeling order. The TA.XT plus texture analyzer (Stable Micro System Ltd., Godalming, Surrey, UK) TPA mode settings were as follows: probe (P/36R), pre-test speed of 2.0 mm/s, test speed of 1.0 mm/s, post-test speed of 2.0 mm/s, and probe compression degree of 50%.

After capturing images of the same batch of samples, the color characteristics of the samples were obtained using traditional methods. The color characteristics values derived from the imaging method were compared with those from the conventional method, establishing a correlational relationship showcasing a high degree of correlation between the two, with a correlation coefficient R^2^ = 0.9684. Therefore, replacing the traditional method of obtaining sample color characteristics with the imaging method proves to be highly feasible. The imaging method conveniently and quickly enhances the color comparison efficiency, and its non-destructive feature, which does not damage the samples, will further reduce experimental costs. Image capture was carried out within a 60 cm × 60 cm × 60 cm light-proof photography studio. The internal top and both sides utilized two shadowless LED lights as light sources, with the incident light angle forming a 45° angle with the samples. A smartphone was affixed to the top to capture sample images. The acquired color images were transmitted to Matlab software on a computer via a USB port for processing and storage.

**Figure 3:**
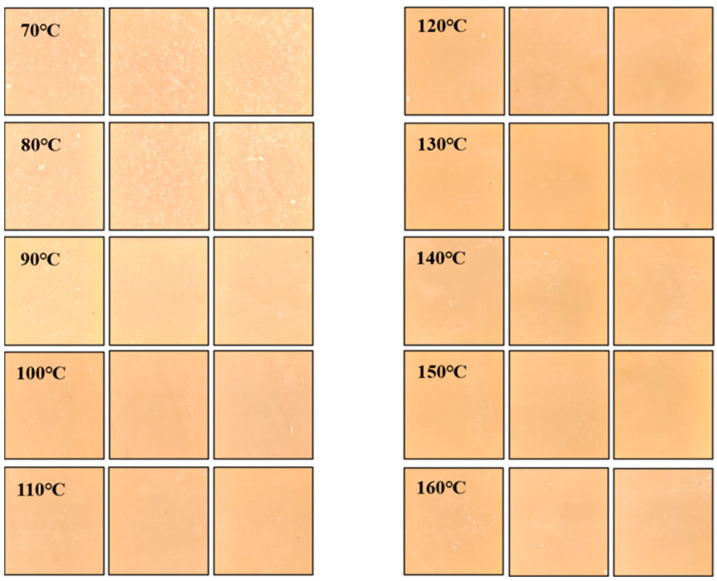
Part of the sample image after preprocessing.

Under the aforementioned sample image capture conditions and environment, images of 12 parallel samples per group were captured and labeled correspondingly, yielding 120 groups of sample images. The captured sample images were preprocessed using Matlab software, with the post-processing image dimensions being 2.5 cm × 2.5 cm at a resolution of 640 × 480. The obtained sample images underwent format conversion within Matlab, transitioning from the RGB format to the Lab format. Subsequently, image features (L*, a*, b*, and contrast) were individually extracted from each sample image. Following the labeling order, the test samples were sequentially subjected to texture TPA mode measurement, from which the real texture characteristic data of 120 groups of samples were obtained and are shown in Figure 3. In order to preserve the accuracy of the predictions, 2 sets of experimental data with evident errors were excluded, and the remaining 118 sample data were selected as the samples for the experimental network prediction model.

### 2.10. Construction of the BP Neural Network

The fundamental algorithms of the BP neural network are the forward propagation of signals and the backward propagation of errors. Inputs undergo non-linear transformations through hidden layer nodes to produce outputs. The computed error between these outputs and the true outputs is measured; if the error is substantial, it initiates the backward propagation of the error, adjusting weights (W) and biases (b) sequentially in reverse. Updated weights and biases yield a new error, steering the error towards gradient descent. This process continues until the error meets minimal criteria or the maximum number of iterations, resulting in a network that, post training, can predict outputs for similar inputs with minimal errors [20,21].

The network environment utilized was MatlabR2022b Software [16]. Based on the images and texture characteristics of the pea protein extrusion texturization product, the parameters for the BP neural network were set as follows: Number of input layer nodes: 4. (Using the L*, a*, and b* image feature values and contrast of the pea protein extrusion texturization product as relevant nodes for the input layer of the BP neural network.) Number of output layer nodes: 1. (For each network training session, one texture feature—hardness, stickiness, chewiness, adhesiveness, resilience, cohesion, or elasticity—was selected for predictive modeling.) Number of hidden layer nodes: selected based on empirical formulas. Learning Rate: 0.01; Minimum Error: 1 × 10^−5^; Maximum Iterations: 1000; Training Set: 90; Test Set: 28.

### 2.11. Particle Swarm Optimization (PSO) and Genetic Algorithm (GA)

The PSO optimization algorithm initializes a group of random particles (each particle having a distinct velocity and position). Each particle adjusts its velocity and position based on shared location data amongst particles, seeking optimal solutions through iterative processes.

Genetic algorithms integrate natural biological evolution theories into problems of searching for optimal solutions in a space. Through selection, crossover, and mutation, they retain good quality characteristics while eliminating unfavorable ones, eventually reaching global optimization through individual genetics.

### 2.12. Evaluation Indicator for the Prediction Model

The MAE (Mean Absolute Error), RMSE (Root Mean Square Error), and MAPE (Mean Absolute Percentage Error) were chosen as accuracy metrics for the prediction model. MAE and MAPE include absolute values in calculations, mitigating the cancellation effect of positive and negative errors. RMSE measures the average error magnitude.

Mean Absolute Error (MAE)

(11) $$MAE=\frac{1}{N}\overset{N}{\underset{i=1}{\sum }}\left|e_{i}\right|=\frac{1}{N}\overset{N}{\underset{i=1}{\sum }}\left|x_{i}-\hat{x_{i}}\right|$$

Root Mean Square Error (RMSE)

(12) $$RMSE=\frac{1}{N}\sqrt{\overset{N}{\underset{i=1}{\sum }}e_{i}^{2}}=\frac{1}{N}\sqrt{\overset{N}{\underset{i=1}{\sum }}{\left(x_{i}-\hat{x_{i}}\right)}^{2}}$$

Mean Absolute Percentage Error (MAPE)

(13) $$MAPE=\frac{1}{N}\overset{N}{\underset{i=1}{\sum }}\left|\frac{e_{i}}{x_{i}}\right|=\frac{1}{N}\overset{N}{\underset{i=1}{\sum }}\left|\frac{x_{i}-\hat{x_{i}}}{x_{i}}\right|$$

### 2.13. Data Analysis

The MatlabR2022b software (Version 9.13) from MathWork was used for preprocessing and extracting image feature values from captured sample images. Microsoft’s Excel 2019 software (Version 16.0) was employed for data analysis, while the Origin data plotting and analysis software (Version 95C, 2018 64 Bit, Origin Lab, Northampton, MA, USA) was utilized for processing measurement data and graphical plotting. IBM’s SPSS Statistics 22 software was used for one-way ANOVA and presented as the average of three determinations with corresponding standard errors. Statistical analysis was conducted using Duncan’s test, and *p*-values < 0.05 were considered statistically significant.

## 3. Results

### 3.1. Impact of Different Processing Parameters on Residence Time

#### 3.1.1. Impact of Screw Speed on Residence Time

**Figure 4:**
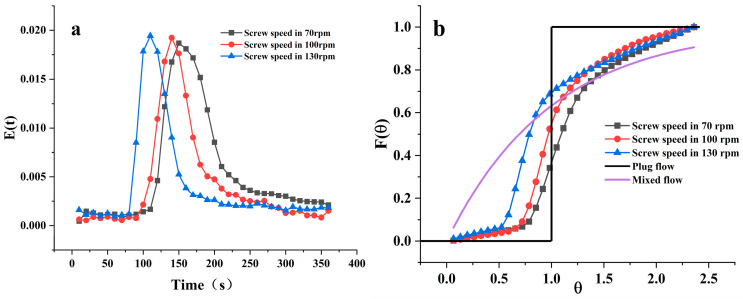
Material Residence Time Distribution E(t) and Cumulative Residence Time Distribution F(θ) at different screw speeds. (**a**) represents the Material Residence Time Distribution E(t) at different screw speeds; (**b**) represents the Cumulative Residence Time Distribution F(θ) at different screw speeds.

As shown in Figure 4a, under the experimental condition of a material feeding speed of 35 rpm, the E(t) curve of different screw speeds is displayed. As the screw speed gradually increases from 70 rpm to 130 rpm, it can be intuitively observed that with the increase in screw speed, the E(t) curve exhibits an overall shift to the left, the width becomes noticeably narrower, and the peak value increases, reducing the distribution range of the material in the barrel. The Mean Residence Time (MRT), variance (σ^2^), and Peclet number Pe under different speed conditions are shown in Table 1 above. The MRT decreases from 186.19 s at a screw speed of 70 rpm to 169.72 s at 100 rpm (a reduction of about 8.8%), and further drops to 152.57 s (a reduction of about 10.1%) when the screw speed reaches 130 rpm, while the variance also reduces from 5823.00 to 3918.88. The increase in screw speed leads to a decrease in both MRT and variance.

The increase in speed concentrates the material distribution in the barrel without being fully dispersed, resulting in lower mixing and a reduction in MRT, which is consistent with the trend presented in the simulation results of Sun et al. [22]. One of the main functions of the screw is to convey the material within the barrel. Nwabueze et al. [23] have analyzed the reasons for the residence time being affected by the screw speed, a higher screw speed provides the material with greater forward force, thereby increasing the material’s velocity in the barrel and reducing the residence time. Dalbhagat et al. [24] explained in a study on rice that the screw speed affects the shear rate as well as the heat exchange in the barrel, leading to a reduction in material viscosity, and thus shortening the residence time. Insufficient heating and incomplete melting affect the texturization degree of the extruded products, further affecting the quality of the extruded products.

Figure 4b shows the F(θ) curve under different screw speed conditions. The flow curve of the material in the barrel is between plug flow and mixed flow; the higher the screw speed, the closer it is to a mixed flow, and the more uniform the material distribution in the barrel. The change in the F(θ) curve is not obvious under different screw speed conditions. From the Pe values in Table 1, it can be observed that under three different screw speed conditions, the Pe number is greater than 10, indicating that during the extrusion process, there is a certain degree of axial mixing of the material in the barrel. However, the lack of significant change in the three groups of Pe values indicates that the screw speed has little effect on the flow model of the material in the barrel, which is also consistent with the trend presented by the F(θ) curve. The screw speed cannot change the flow state of the material in the barrel but can determine the flow speed, i.e., the residence time of the material in the barrel. Similar research conclusions also appeared in Sisay’s study [25].

#### 3.1.2. Impact of Different Material Feeding Speeds on Residence Time

**Figure 5:**
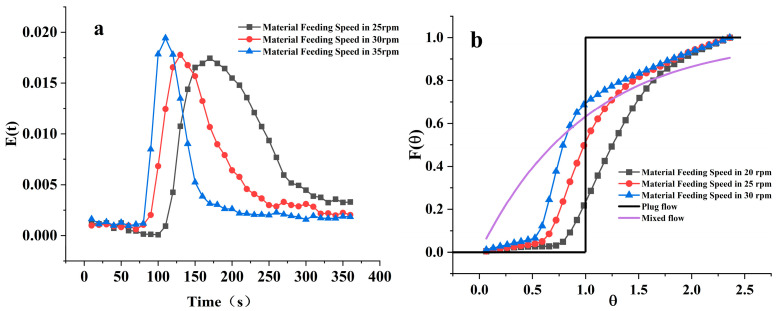
Material Residence Time Distribution E(t) and Cumulative Residence Time Distribution F(θ) at different material feeding speeds. (**a**) represents the Material Residence Time Distribution E(t) at different material feeding speeds; (**b**) represents the Cumulative Residence Time Distribution F(θ) at different material feeding speeds.

Figure 5a shows the E(t) curve of different material feeding speeds under the experimental condition of a screw speed of 130 rpm. As the material feeding speed increases from 25 rpm to 35 rpm, the trend of the E(t) curve changes in a manner quite similar to that of the screw speed, showing a general leftward shift, narrower width, and an increase in the peak value. The increase in material feeding speed also shortens the residence time of the material in the barrel, reducing the distribution range. The Mean Residence Time (MRT), variance (σ^2^), and Peclet number (Pe) are shown in Table 2. The MRT decreases from 204.09 s at 25 rpm to 173.52 s at 30 rpm (a reduction of about 14.97%), and further drops to 152.57 s (a reduction of about 12.07%) when the material feeding speed reaches 35 rpm, with the variance also reducing from 4871.29 to 3918.88. Similar to the increase in screw speed, the increase in material feeding speed leads to a decrease in both MRT and variance.

The material feeding speed determines the fill degree of the material within the barrel; when the screw speed remains constant, a higher material feeding speed results in a greater fill degree within the barrel, filling the barrel space faster and increasing the amount of material within the barrel over the same time period. The material filling the entire barrel augments the forward flow impetus of the material, synchronizing positively with the screw speed to achieve a higher flow velocity, ultimately shortening the residence time of the material within the barrel.

Figure 5b shows the F(θ) curve under different material feeding speed conditions. As the material feeding speed increases, the F(θ) curve approaches plug flow, reducing the material distribution within the barrel and decreasing the mixing degree of the material. Hence, increasing the material feeding speed is actually detrimental to material mixing. As can be discerned from the Pe values in Table 2, the material feeding speed significantly impacts the Pe value, which increases with the material feeding speed, which aligns well with the trend presented by the F(θ) curve. The substantial difference between the different material feeding speeds, compared to the difference in the screw speed, illustrates that although increasing both screw speed and material feeding speed can reduce the material’s residence time within the barrel, the mechanisms and principles influencing the residence time are different. The increase in material feeding speed can significantly shorten the material’s residence time but reduces the degree of mixing of the material. In practical processing production, it is essential to adjust for an appropriate material feeding speed to ensure adequate mixing and residence time of the material within the barrel to achieve optimal product quality.

Upon comparison, it is observed that the material feeding speed has a more pronounced effect on MRT and σ^2^ compared to the screw speed. Therefore, the material feeding speed is a highly noteworthy operational parameter during extrusion processing, as the fill degree of material within the barrel not only determines the residence time but also affects the mixing degree and flow state of the material.

#### 3.1.3. Impact of Different Initial Material Moisture Contents on Residence Time

**Figure 6:**
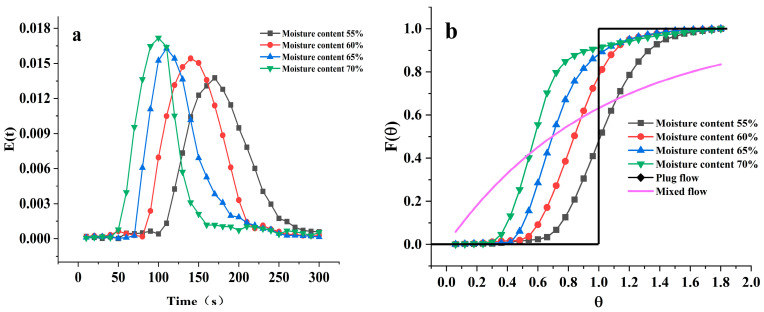
Material Residence Time Distribution E(t) and Cumulative Residence Time Distribution F(θ) at different initial material moisture contents. (**a**) represents the Material Residence Time Distribution E(t) at different initial material moisture contents; (**b**) represents the Cumulative Residence Time Distribution F(θ) at different initial material moisture contents.

Figure 6 shows the E(t) curves under different initial material moisture content at a selected screw speed of 100 rpm and material feeding speed of 30 rpm, based on the earlier experimental results. As the initial material moisture content gradually increases from 55% to 70%, compared to the effects of screw speed and material feeding speed, the impact is more significant. With the increase in initial material moisture content, the E(t) curve collectively shifts leftward, narrows in width, and the peak value increases, indicating that as the initial material moisture content rises, the residence time of the material in the barrel shortens, and the distribution range decreases. The Mean Residence Time (MRT), variance (σ^2^), and Peclet number (Pe) are shown in Table 3. With the rise in moisture content, both MRT and σ2 notably decreased, the residence time of the material in the barrel shortened from 265.2 s to 166.6 s, and the variance also decreased from 1913.60 to 1338.97, leading to a more concentrated material distribution, which was unfavorable for material mixing.

The decrease in the average amount of time a substance stays in a particular environment at increased levels of moisture is linked to the lubricating influence of higher levels of moisture, which aligns with Hoyos-Concha et al.’s [26] discoveries on fish extrudate. Whereas moisture lowers the viscosity of the molten material, high temperature promotes moisture evaporation, forming a saturated steam micro-environment within the extrusion barrel. Under this environment, the friction between the material and the screw further decreases. The barrel, possessing high-energy water molecules and steam, facilitates smoother material flow, simultaneously opening the protein molecular chains and re-aggregating to form a structured composite. When the initial material moisture content decreases, most of the water participates in the denaturation reaction of the protein, leaving less water to reduce friction and enhance lubrication. Hence, the material flow is restricted, leading to an extended residence time. Similar research results have explained that an increase in initial material moisture content reduces the thrust needed for the melt to pass through the barrel, simultaneously reducing the friction between the material and the screw, the material and the barrel, and the screw and the barrel.

Figure 6b presents the F(θ) curves under different initial material moisture content conditions. With the increase in moisture content, the F(θ) curve approaches plug flow, reducing material distribution within the barrel and diminishing the degree of material mixing within the barrel, thus increasing the initial material moisture content is detrimental to material mixing. From the Pe values in Table 3, it was evident that moisture content significantly impacted the Pe value, and as the moisture content rose, the Pe value increased, which aligned well with the trend exhibited by the F(θ) curve, although with considerable differences between different moisture content conditions in the F(θ) curve. An increase in moisture content could significantly shorten the material’s residence time and decrease the material’s degree of mixing, similar findings were also observed in Sisay’s research on the extrusion of low gluten wheat [25]. In actual production, it was necessary to adjust to both the optimal material feeding speed and optimal moisture content according to the relationship between the product’s quality requirements and the machine’s efficiency.

### 3.2. Microstructure of Extruded Pea Protein Products at Different Residence Times

The shear force determines the magnitude of shear action, serving as a critical factor in promoting physicochemical reactions. The energy input into the system provides the energy source for the entire reaction. By examining the microstructural changes in the extruded products, this study explores the integrated action model of system parameters under different processing parameter conditions.

**Figure 7:**
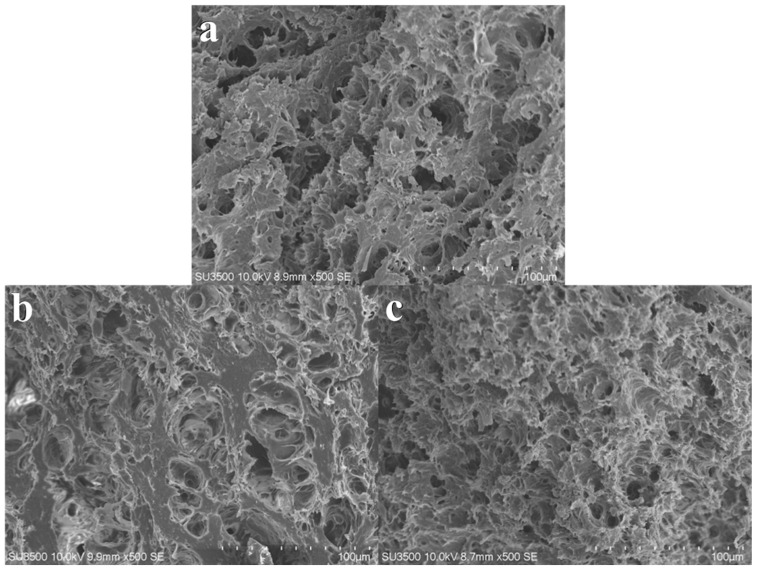
Microstructure of texturized products at different screw speeds. (**a**) represents the screw speed at 70 rpm; (**b**) represents the screw speed at 100 rpm; and (**c**) represents the screw speed at 35 rpm. Material feeding speed at 30 rpm.

**Figure 8:**
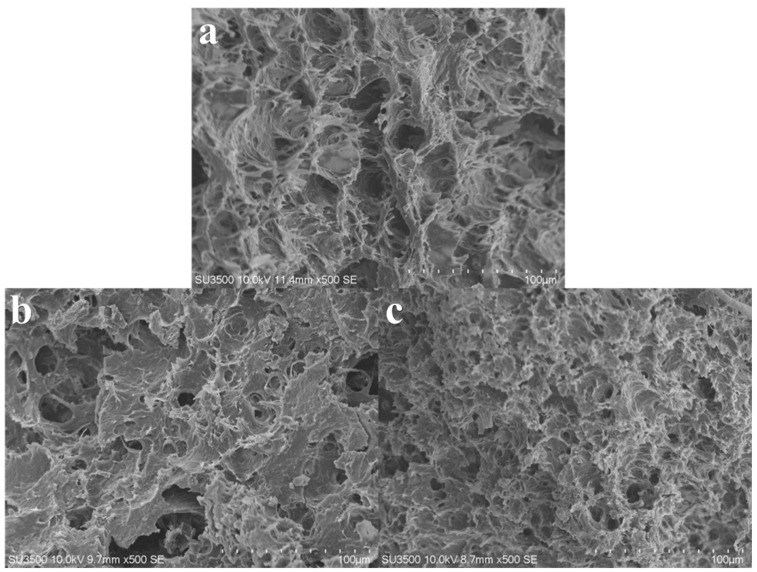
Microstructure of texturized products at different material feeding speeds. (**a**) represents material feeding speed at 25 rpm; (**b**) represents material feeding speed at 30 rpm; and (**c**) represents material feeding speed at 35 rpm. Screw speed at 100 rpm.

**Figure 9:**
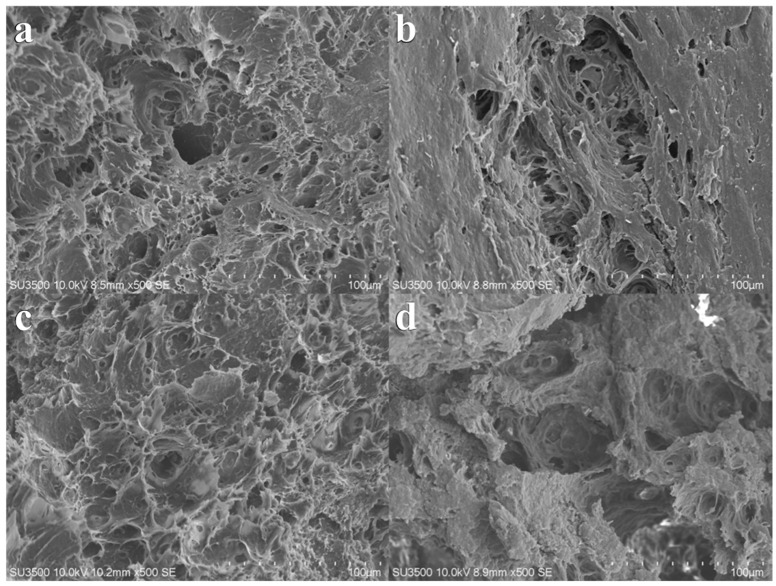
Microstructure of texturized products with different initial material moisture. (**a**) represents the initial material moisture at 55%; (**b**) represents the initial material moisture at 60%; (**c**) represents the initial material moisture at 65%; and (**d**) represents the initial material moisture at 70%. Screw speed at 100 rpm; material feeding speed at 30 rpm.

Figure 7 depicts significant differences in the microstructure of the extruded products under varying screw speed conditions. At a screw speed of 70 rpm, the extruded products appear loose and unconsolidated with a coarse overall microstructure featuring numerous and larger pores. With the increase in screw speed, the extruded products become denser, and compared to low-speed conditions, their microstructure displays fewer pores, finer protein particles, and a more compact distribution. Figure 8 demonstrates that an increase in material feeding speed also impacts the final microstructure of the extruded products. Higher material feeding speeds result in a larger amount of material in the barrel, enhancing the compressive and shear actions between the materials, and between the materials and the screw, thereby yielding products with smaller microstructural voids and denser structures. Moisture content determines the flow and mixing state of the materials in the barrel. Figure 9 demonstrates that higher moisture content enhances material fluidity, shortening the residence time within the barrel. Consequently, the microstructure shows less evident agglomeration of protein particles, and as moisture content increases, the structure becomes increasingly sparse, leading to a reduction in hardness.

### 3.3. Prediction and Analysis of Product Texture Characteristics Based on PSO/GA-BP Neural Network and Image Analysis

The total sample size for the network prediction model was 118. To comprehensively characterize the texture features of the extruded products, two sets of experimental data with apparent errors were removed when measuring the texture indexes of the extruded products to avoid affecting the accuracy of the prediction. To test the accuracy of the neural network prediction model, the 118-sample data set was divided into a training set and a test set, with 90 in the training set and 12 in the test set. The neural network output the trained prediction model from the 90-sample training set. The 12-sample test set data were input into the prediction model to obtain the predicted values, which were then compared with the real values of the test set samples.

#### 3.3.1. Prediction Deviation in Optimized Model

**Figure 10:**
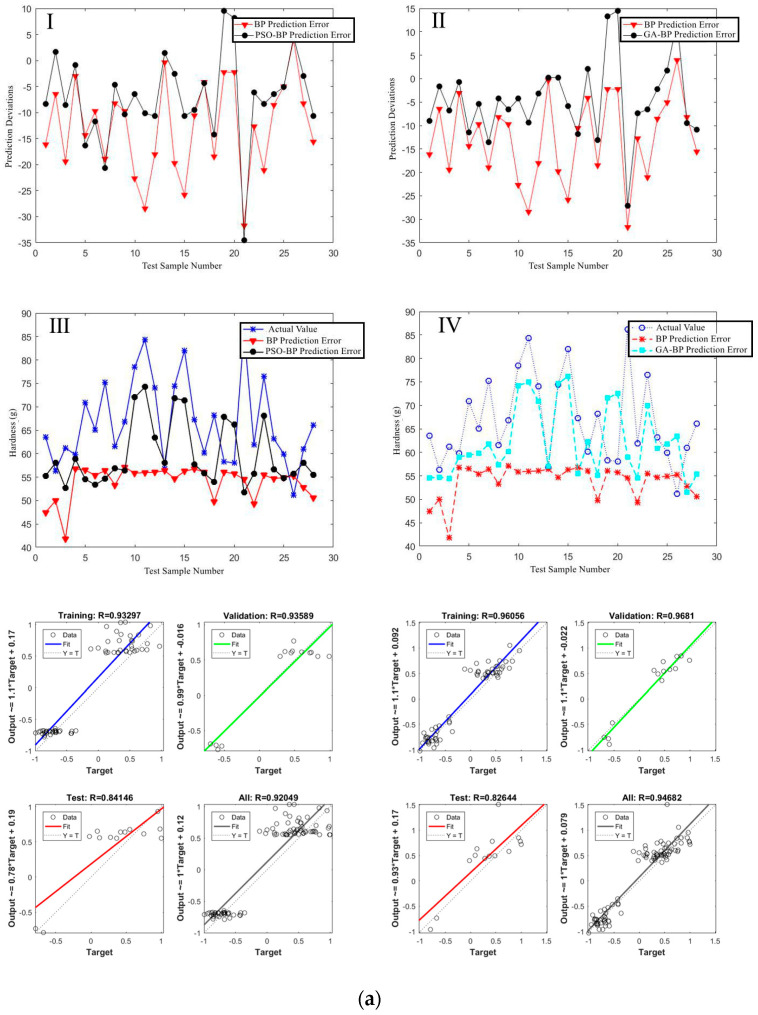

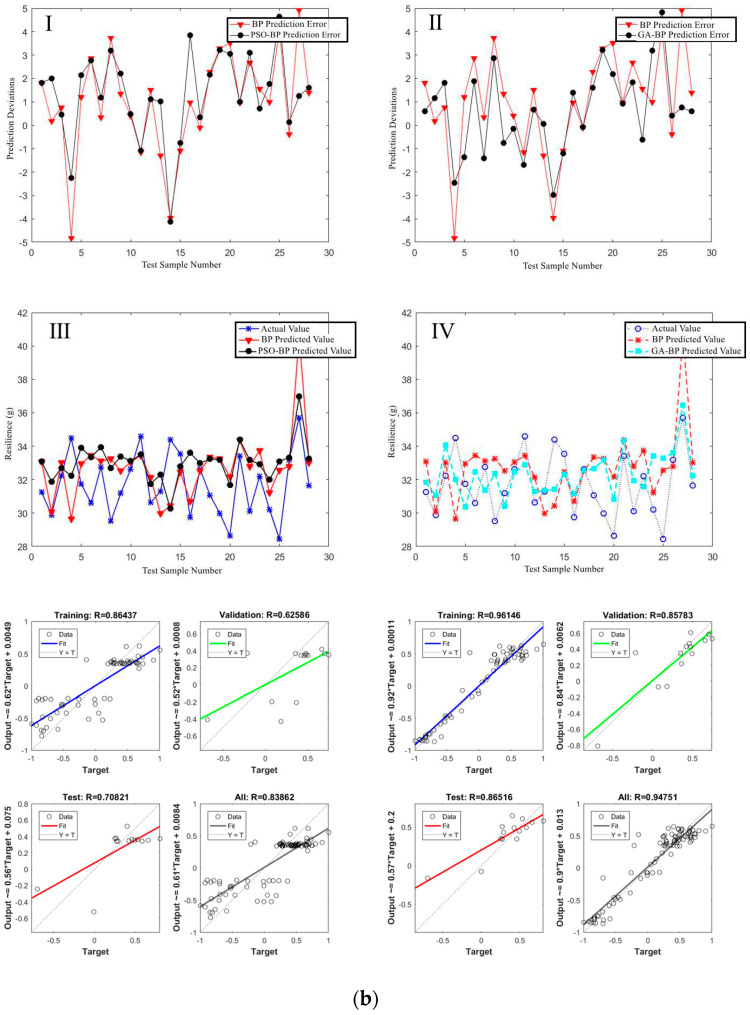

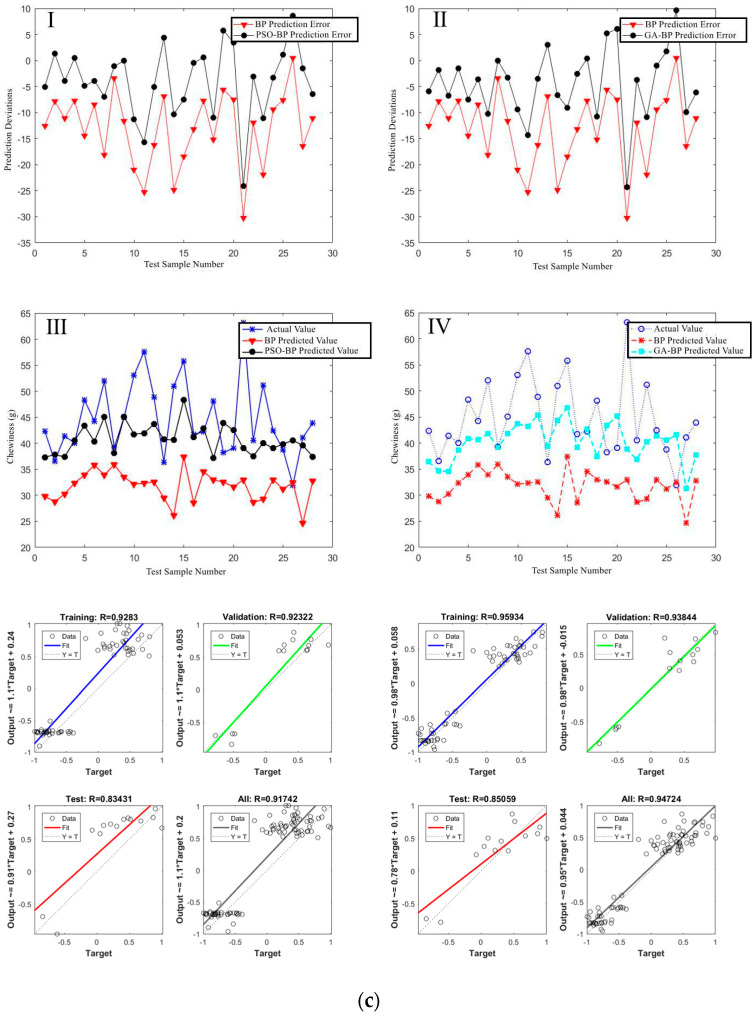
Comparison of the prediction deviations of texture characteristic prediction models. (**a**) represents the prediction deviations of Hardnes; (**b**) represents the prediction deviations of Resilience; (**c**) represents the prediction deviations of Chewiness. (In (**a**–**c**), “I” represents the comparison between the BP prediction error and the PSO-BP prediction error for “Hardness”, “Re-silience” and “Chewiness”; “II” represents the comparison between the BP prediction error and the GA-BP prediction error for “Hardness”, “Resilience” and “Chewiness”; “III” represents the comparison between the BP predicted value and the PSO-BP predicted value with the actual value for “hardness”, “resilience” and “chewiness”; “IV” represents the comparison between the BP predicted value and the GA-BP predicted value with the actual value for “Hardness”, “Resilience” and “Chewiness”. The lower part of (**a**–**c**) respectively represents the comparison of the correlation coefficients between PSO and GA predictions for “Hardness”, “Resilience”, and “Chewiness”).

Figure 10 shows the comparison of the prediction deviations before and after optimization in the texture prediction model, with sub-Figure 10a–c representing hardness, resilience, and chewiness. All instances of Ⅰ and Ⅲ in Figure 10a–c show the prediction deviations between the predicted values and actual values before and after Particle Swarm Optimization (PSO) optimization, while Ⅱ and Ⅳ show the deviations after Genetic Algorithm (GA) optimization. From the deviation graphs, it can be deduced that the deviation between the predicted values and actual values significantly reduces after optimization with both algorithms, thus better aligning the post-optimization predicted value curves with the actual value curves.

To accurately measure the degree of model fit, we conducted an analysis of the correlation coefficients for various algorithms. Based on Figure 10, the hardness, resilience, and chewiness of the extrudates are highly correlated with the image features (L*, a*, b* and contrast) of the extruded products, with a correlation coefficient greater than 0.94. During the extrusion process, as the extrusion temperature increases under a high-energy input environment, the protein molecular chains open to a more thorough degree within the barrel. The cross-linking and reorganization of the molecules increase, and the higher-energy water molecules become more active at higher temperatures, enhancing the plasticizing effects. The mobility of the protein’s primary structure fragments is enhanced, which facilitates molecular chain stretching and alignment [27,28]. The shear and mixing actions of the screw cause the protein molecular alignment to become more compact, increasing the density of the protein melt, thereby enhancing the hardness and chewiness of the extrudates, with a consequent change in the products’ resilience [29,30]. In the energy input environment of this experiment, the color of the extruded products generally presents as brownish-yellow. As the extrusion temperature continually rises, the color of the extruded products deepens. This phenomenon specifically manifests in the product’s image features as an overall richness in yellow hue, less green hue, and a gradual reduction in brightness (a* < 0, b* > 0, L* gradually decreases). Simultaneously, the hardness of the extruded products gradually increases due to the promotion of protein melting in the barrel with rising temperature, reducing viscosity, and thus extending the residence time [31] in the barrel and prolonging the Maillard reaction time [32], which deepens the browning degree of the extruded products. This illustrates a mechanistic correlation between the image features and the hardness of the extruded products. The resilience of the extruded products is directly affected by the gel content and hardness, and to some extent, the L* of extruded products reflects the amount of gel content, with a higher gel content leading to more reflected light and greater overall brightness, suggesting an indirect correlation between the resilience of extruded products and their image features. The chewiness of the extruded products is also directly affected by factors such as hardness and resilience. Therefore, a rational prediction of the texture characteristics of the extruded products can be made through the color changes of the extruded products.

#### 3.3.2. Prediction Model Error

The prediction deviation values and comparison between the predicted values and actual values from the PSO/GA-BP neural network for texture characteristics reveal that the prediction deviations of the optimized BP neural network have been reduced, rendering the predicted values closer to the actual values, which is also reflected in the prediction error. As shown from the Table S1, the prediction error of the optimized model in terms of MAE, MSE, RMSE, and MAPE has been reduced, which is consistent with the trend in prediction deviation values. The errors in the prediction model optimized by the GA optimization algorithm are all lower than those optimized by the PSO optimization algorithm. This indicates that in the BP neural network based on image features, the initial weights and thresholds obtained through optimization via the GA optimization algorithm enable the BP neural network to better find the global optimal solution [33].

#### 3.3.3. Prediction Model Correlation Coefficients

The experiment demonstrates that both PSO and GA optimization algorithms can reduce prediction errors, enhance prediction accuracy, and avoid the risk of the BP neural network algorithm getting trapped in local optima. Table 4 below shows the comparison of the correlation coefficients of the two optimization methods and the prediction model before optimization. The critical correlation coefficient for this experimental sample environment at a significance level of *p* = 0.05 is 0.374. The calculated values exceed the critical correlation coefficient value. Comparative analysis reveals that the GA optimization algorithm is superior overall to the PSO optimization algorithm. The correlation coefficients of the prediction model optimized by the GA optimization algorithm are higher than those optimized by the PSO optimization algorithm in all texture characteristics except hardness. The table data indicate a significant correlation between the actual values of various textural properties (including hardness, chewiness, and resilience) of the extruded products and the PSO neural network prediction model’s predicted values. This allows for rapid and precise predictions of texture characteristics through the color information of extruded product images using neural networks, providing the possibility of rapid, online, and precise detection.

## 4. Conclusions

This paper employed pea protein extrusion texturization experiments as the medium; by setting different processing parameter conditions such as screw speed, material feeding speed, and initial material moisture content, the Residence Time Distribution (RTD) of the material within the extruder barrel under different parameters was ascertained using the tracer method. Additionally, image colorimetry was employed to analyze the variation trends of the E(t) and F(t) curves under different parameter conditions. The microstructure characterization of extruded products under different parameter conditions was carried out using Scanning Electron Microscopy (SEM), analyzing the impact of various variables on texture indicators. Finally, a texture quality prediction model based on a BP neural network and the image analysis of extruded products was established, and PSO and GA optimization algorithms were employed to optimize the neural network, to prevent the network from falling into local optima, and to establish the correlation between the image features and texture quality. The main research conclusions are as follows:

The results indicate that the RTD of materials decreases with the increase in screw speed, feeding speed, and initial moisture content. In terms of residence time, the effect of the material feeding speed is the most significant, followed by screw speed; similarly, in terms of the degree of material mixing, the impact of the material feeding speed is also more significant than that of screw speed. The level of moisture content directly affects the quality of extruded products.

Different processing parameters have different impacts on the microstructure of the extruded products. Under different screw speeds, the microstructures of the extruded products exhibit significant differences.

The experimental results show that the prediction effect of the GA optimization algorithm model is better than the PSO model, with the prediction accuracy and correlation coefficient also significantly better than the PSO optimization algorithm. Through the prediction model correlation coefficients, it was found that the hardness, chewiness, and resilience textural characteristics of the extruded products all had a strong correlation with the image features of the extruded products.

In this study, a texture quality prediction model was developed using BP neural networks and extruded product images. Image features and contrast were used as input layers for texture quality prediction. The correspondence between image features and specific texture indices can be further explored in future studies, and a rational explanation in terms of processing parameters and energy input can be provided.

## Figures and Tables

**Table 1:** Residence time parameters for different screw speeds.

Screw Speeds (rpm)	MRT (s)	$\sigma^{2}$ ($s^{2}$)	Pe
70	186.19 ± 2.83 ^a^	5823.00 ± 176.48 ^a^	10.80 ± 0.46 ^a^
100	169.72 ± 1.67 ^b^	4569.29 ± 151.82 ^b^	11.48 ± 0.21 ^a^
130	152.57 ± 5.85 ^c^	3918.88 ± 126.94 ^c^	10.74 ± 0.38 ^a^

Note: Different letters ^a–c^ in the same column indicate significant differences between groups (*p* < 0.5).

**Table 2:** Residence time parameters for different material feeding speeds.

Material Feeding Speed (rpm)	MRT (s)	$\sigma^{2}$ ($s^{2}$)	Pe
25	204.09 ± 3.48 ^a^	4871.29 ± 67.59 ^a^	10.80 ± 0.31 ^c^
30	173.52 ± 1.59 ^b^	4158.80 ± 34.61 ^b^	13.41 ± 0.46 ^b^
35	152.57 ± 0.94 ^c^	3918.88 ± 16.58 ^c^	15.98 ± 0.17 ^a^

Note: Different letters ^a–c^ in the same column indicate significant differences between groups (*p* < 0.5).

**Table 3:** Residence Time Parameters for different initial material moisture contents.

Initial Material Moisture Content (%)	MRT (s)	$\sigma^{2}$ ($s^{2}$)	Pe
55	265.21 ± 4.76 ^a^	1913.60 ± 37.26 ^a^	40.15 ± 0.68 ^d^
60	209.69 ± 3.18 ^b^	1598.00 ± 28.64 ^b^	44.14 ± 1.24 ^c^
60	181.75 ± 3.05 ^c^	1440.86 ± 41.93 ^c^	54.35 ± 0.88 ^b^
70	166.62 ± 2.49 ^d^	1338.97 ± 18.52 ^d^	69.90 ± 3.06 ^a^

Note: Different letters ^a–d^ in the same column indicate significant differences between groups (*p* < 0.5).

**Table 4:** PSO/GA-BP neural network prediction model correlation coefficient.

Texture Characteristics	BP Prediction Model	PSO-BP Prediction Model	GA-BP Prediction Model
Hardness	0.92049	0.94464	0.93913
Resilience	0.91864	0.93186	0.94040
Chewiness	0.91742	0.94677	0.94724

## Data Availability

Data is contained within the article.

## Supplementary Materials

The following supporting information can be downloaded at: https://www.mdpi.com/article/10.3390/foods12244408/s1, Table S1: PSO/GA-BP Neural Network Prediction of Texture Characteristic Error.
