# Supplementary material for: Study on the Residence Time and Texture Prediction of Pea Protein Extrusion Based on Image Analysis
Source: Foods. 2023 Dec 7;12(24):4408. doi: 10.3390/foods12244408 (PMC10742850; doi:10.3390/foods12244408)
Supplement: Supplementary file 1 [file foods-12-04408-s001.zip › foods-2743879-supplementary.pdf]

Supplementary File

Table S1:

Table S1. PSO/GA-BP Neural Network Prediction of Texture Characteristic Error

Texture Characteristics	Algorithm		MAE	MSE	RMSE	MAPE
Hardness	PSO	A	13.7506	271.2393	16.4693	19.2860%
		B	8.2494	109.4865	10.4636	11.6206 %
	GA	A	9.9642	177.0484	13.3060	13.6932 %
		B	7.1850	88.2578	9.3946	10.5262 %
Resilience	PSO	A	2.2198	7.3889	2.7183	7.2309 %
		B	1.4304	3.4231	1.8502	4.6031 %
	GA	A	1.9160	5.6788	2.3830	6.0788 %
		B	1.7344	4.3140	2.0770	5.6107 %
Chewiness	PSO	A	9.3944	136.6132	11.6882	19.2639 %
		B	5.6697	49.1289	7.0092	12.2022 %
	GA	A	13.0594	218.4931	14.7815	27.6970 %
		B	5.7408	64.5064	8.0316	12.2837 %

Note: A represents before optimization; B represents after optimization.
